# Where best practice pain care and patient expectations for care meet: Exploring patient expectations around chronic pelvic pain, physiotherapy, and the biopsychosocial model of care

**DOI:** 10.1177/17455057251349626

**Published:** 2025-06-25

**Authors:** Michelle Hong, Allison Crone, Elza Gashi, Meghan Pietluch, Maddy Reinders, Jayden Uchida, Adriano Nella, Crystal MacKay, Olivia Drodge, Rachael Bosma

**Affiliations:** 1 Department of Physical Therapy, University of Toronto, ON, Canada; 2 Women’s College Hospital, Toronto Academic Pain Medicine Institute, ON, Canada

**Keywords:** chronic pelvic pain, patient expectations, pelvic health physical therapy

## Abstract

**Background:**

Pelvic physiotherapy (PT) is a holistic and evidence-based treatment for chronic pelvic pain (CPP). It is important to understand patient expectations for treatment to improve patient satisfaction and outcomes. The current literature lacks information about patient expectations for CPP pelvic PT.

**Objectives:**

To describe the expectations around treatment and the role of pelvic PT for patients with CPP.

**Design:**

We conducted a qualitative study and interviewed 10 participants who were on the waitlist for CPP PT at Women’s College Hospital in Toronto, ON, about CPP and pelvic PT.

**Methods:**

We recruited patients on the pelvic pain PT waitlist who were assigned female at birth, 18 years of age or older, diagnosed with CPP for more than 6 months. The 1 -h long interviews were conducted via Zoom by two PT students before being transcribed with NVivo. Inductive content analysis was used to create themes and categorize the participant data.

**Results:**

We describe three main themes to convey the experiences of participants living with CPP and their expectations for pelvic PT: (1) Expectations are clouded by a lack of understanding, (2) Pelvic PT will provide a new way to get relief, and (3) My role is to be open to try new things.

**Conclusions:**

Pelvic PT should incorporate education regarding CPP, strong therapeutic alliance with the patient, effective communication, and integration of the biopsychosocial approach to care to better meet patient expectations and improve quality of care. This study highlights the critical importance of providing patients with consistent, accurate, and comprehensive education on CPP, pain treatment and self-management strategies, and the role of pelvic PT. By delivering this foundational knowledge early in the patient’s treatment plan, we can influence patient expectations, enhance both patient engagement and outcomes in pelvic PT, leading to a more holistic, informed, and effective approach to patient care.

## Introduction

Chronic pelvic pain (CPP) is a debilitating condition that affects approximately 15% of cisgender women worldwide.^1^ It is characterized by pain originating from organs and/or structures in the pelvic region that lasts more than 3 months.^2^ CPP is associated with dysfunction in multiple body systems, including urinary, sexual, bowel, myofascial, and gynecological/urological systems, which consequently has drastic impacts on healthcare costs, referrals to healthcare services, and their own quality of life.^1^ Various theories suggest potential causes for different CPP conditions; however, the precise pathophysiology remains unclear for many patients.^3^ Approximately 50% of CPP cases present with overlapping symptoms of other conditions, such as irritable bowel syndrome, complicating the clinical picture and increasing the risk of misdiagnosis.^4^ Furthermore, women with CPP often experience negative healthcare interactions due to stigma, leading to increased emotional toll, decreased trust, reluctance to report symptoms, further delaying, and/or receiving appropriate treatment and diagnosis.^3,5^ Due to the complex interplay between the physical and psychological comorbidities in individuals with CPP, evidence supports a multidisciplinary treatment approach, including pelvic physiotherapy (PT), to manage specific patient factors.^2,6^

Pelvic PT has widely been recognized as an evidence-based treatment for individuals with varying pelvic conditions.^7^ Pelvic physiotherapists have specialized training and are rostered with their Regulatory College to perform internal assessment and treatment of conditions within the pelvis such as CPP, urinary and fecal incontinence, pelvic organ prolapses, sexual dysfunction, and pregnancy and postpartum-related problems.^8–10^ Evidence-based pelvic PT employs a biopsychosocial approach to care.^7^ This holistic approach allows for a comprehensive assessment to identify and target the underlying drivers of patient-reported symptoms.^7^ Despite this wide and valuable skillset, pelvic PT is not well understood by the public. In Kasawara et al.’s^11^ study about public knowledge of PT treatment for urogynecology dysfunction, 50% of individuals did not know about its role. Many individuals are unaware of the specialty of pelvic PT and mistakenly associate it with traditional orthopedic PT. This lack of awareness negatively affects patient expectations for pelvic PT, consequently influencing treatment outcomes.

Patient expectations are defined as a belief that something will happen in the healthcare context based on a combination of cognitive and affective components, including values, experiences, emotions, and social norms.^12,13^ Studies have demonstrated a link between positive health outcomes and increased patient satisfaction when clinicians incorporate patient expectations into clinical care settings, such as PT treatment.^12–15^ In 2016, Testa and Rossettini^15^ reported that patient expectations and prior experiences about PT treatment significantly influence a patient’s pain experience and therapeutic outcome. Despite these findings, previous research has indicated that patient expectations are infrequently discussed and underestimated in physical therapy practice.^16,17^ In a 2021 qualitative study exploring patient expectations of PT for low back pain, Unsgaard-Tønsdel and Søderstrøm^18^ found that prior to treatment, patient expectations were concentrated on exercises and a diagnosis, whereas patient expectations 6 months after treatment focused on self-management.^18^ This highlights a need for healthcare professionals to continuously discuss patient expectations throughout care to prevent a disconnect between expectations and treatment in order to provide quality care.

While evidence supports the need to incorporate patient expectations into chronic pain management, there are limited studies addressing the treatment expectations held by CPP patients regarding pelvic PT. Given the complexities of CPP, the sensitive nature of this area of the body, and the higher prevalence of stigma and trauma, little is known about patient experiences and expectations surrounding pelvic PT.^3^ It is unclear whether CPP patients are aware of the multi-modal nature of pelvic PT treatment, and therefore, their expectations may misalign with intended outcomes, leading to poor prognosis. Assessing CPP patients’ expectations regarding their care is crucial for fostering an understanding of the multifaceted nature of their pain and the importance of active treatment participation. Incorporating patient expectations into clinical practice enhances patient-reported outcomes and pain self-efficacy, thereby strengthening the therapeutic relationship.^19^ Leveraging a qualitative research approach, the aim of this study was to explore the perspectives of patients regarding their understanding and expectations around pelvic PT for CPP management.^20^ Given the important relationship between expectations and health outcomes, the results of this study have direct relevance for clinical practice.^19^

## Methods

### Study design

We conducted a qualitative study using semi-structured interviews to understand patients’ perceptions of CPP and their expectations regarding pelvic PT. The results were analyzed using inductive content analysis to generate themes from participant responses with no prior construct. The reporting of this study conforms to the Consolidated Criteria for Reporting Qualitative Research statement.^21^ This research was approved by the Women’s College Hospital (WCH) Research Ethics Board (REB# 2023-0050-E) and the University of Toronto Health Sciences Research Ethics Board (REB# 45660) in Ontario, Canada.

### Setting

We conducted this research at the Toronto Academic Pain Medicine Institute (TAPMI) at WCH in Toronto, ON, Canada. TAPMI is an interdisciplinary network of five hospitals that provide care for individuals living with chronic pain by addressing the multidimensional factors (e.g., psychosocial, environmental, and physical) that contribute to chronic pain.

### Research team

Our research team included six Master of Science in Physical Therapy students from the University of Toronto (AC, EG, MH, MP, MR, JU, and CM), a physiotherapist faculty (CM), a pelvic physiotherapist (OD), a chronic pain scientist (RB), and a research coordinator (AN).

### Participants

Participants were asked to participate in this study if they were on the wait list for pelvic PT at WCH and met the following inclusion and exclusion criteria: (1) Female assigned at birth, (2) 18 years of age or older, (3) diagnosed with CPP (pain for >6 months) as determined by their referring provider, participants were excluded if they (1) did not have access to a compatible technology (smartphone, tablet, computer/laptop) for a video or telephone interview and (2) can communicate in English.

### Recruitment

Convenience sampling was utilized to recruit participants from WCH. Participants were recruited the following procedure: (1) a physiotherapist within the patients’ circle of care informed potential participants about the research opportunity, (2) the physiotherapist received verbal consent to share their contact information with the research team, (3) patients were contacted via telephone by the research team to outline the details of the study and determine if the patient meets the inclusion criteria, (4) a Zoom interview was scheduled with eligible patients interested in participating and verbal consent was obtained prior to the interview. Verbal consent was approved by the WCH REB and the University of Toronto Health Sciences REB. The consent form was read to participants during the interview process, and consent was recorded alongside the interview. This reduced burden on the participants while meeting ethical standards for fully documented informed consent. Participants were informed that all participation was optional, would not influence the care they received, and that they could withdraw from the study at any time. No participants withdrew from the study. Formal sample size was not determined a-priori, rather, as data were analyzed, we determined the saturation of the coding framework across transcripts.^22^

### Data collection

The research team developed a semi-structured interview guide consisting of eight open-ended questions rooted in our research study objectives (Supplemental material). Interview question examples include: (1) What do you think is going to happen during your pelvic PT appointment and (2) What is your role in your PT care? Ten interviews were held on Zoom between January and April 2024 by two female research team members (MP, MR). The students received formal training during the second year of their education program and additionally conducted two, 1-h long practice interview sessions to further develop their interviewing techniques. The participants had no prior relationship with the researchers conducting the interview. Only one member of the research team led the interview by asking the primary interview questions and probing questions when necessary to supplement participants’ responses. The second team member took field notes during each interview to capture significant emotions and impressions (e.g., tone of voice, expressions, hand gestures) to further contextualize the conversation for data analysis. The primary and secondary interviewer would switch roles for each interview, but only the primary interviewer would ask questions, and the secondary interviewer would not intervene. To mitigate any power imbalances that may have arisen due to having two researchers present during the interview, the interviewer took extra focus on providing a comforting and safe space and informing participants multiple times that they can skip questions or drop out of the study at any time. Each interview was audio-recorded, transcribed verbatim using NVivo, and checked for accuracy by two other research team members in Microsoft Word (AC, JU). A demographic questionnaire was administered prior to each interview to capture participant characteristics such as age, gender identity, socio-economic status, education, transportation needs, and healthcare services used in the past. Interviews were scheduled for 1 h and all participants completed the interview within 1–2 weeks after enrollment.

### Data analysis

We analyzed the data using inductive content analysis, a method to identify, analyze, and report patterns within data when there is not an underlying theoretical construct and when the results are expected to inform practice.^23^ All research team members reviewed the first three interview transcripts to create a preliminary coding scheme. The research team then met to finalize a master code book by grouping similar codes to create parent codes (main codes) with subcategory of the code underneath and ensuring that there were no repeat codes. Each interview transcript was coded independently by two members of the research team who then met to discuss and reach a consensus for the coding of each transcript. The team reviewed the suggested codes and co-created a preliminary coding list by grouping similar codes to capture as many aspects of the content as possible. Coding saturation was determined to be reached when new codes were not added from the narrative of successive interviews and sufficient context was established to describe the perspectives of individuals with CPP. Once code saturation was reached in principle, the identified codes were applied to the remaining transcripts independently. Upon the completion of coding, the research team met to discuss themes among the codes. Parent codes were grouped under appropriate themes and excerpts from transcripts were selected to support each theme. Themes were refined to best represent the data and ensured they supported our study objectives. Analysis was considered complete once any inconsistencies were resolved and consensus was achieved.

### Rigor

The fundamental qualities of rigor were outlined by Lincoln^24^ and encompass credibility, dependability, and confirmability. Through the application of the rigorous approach described by Higginson et al.,^25^ the team ensured credibility by meticulous verification of our data across all stages of collection and analysis. This process resulted in an accurate representation of our study findings, supported by evidence that connects our results with existing literature. Comprehensive details regarding our sample and study context facilitated transferability. For dependability, we documented our methodology in thorough detail, ensuring transparency and enabling replication of our study. Confirmability was enhanced by engaging in reflexive practices throughout data collection and analysis, including reflexive dialogues among team members and sharing of field notes. Our research team was comprised of experienced researchers (more than 10 years), a clinician (11 years), and trainees. The clinician team member brought valuable expertise and potential preconceptions based on their experience with the population. Throughout the data interpretation process, we continually questioned preconceived notions and maintained flexibility when moving between raw data and content analysis to ensure alignment.

## Results

Ten participants met eligibility and consented to the interview. Participant age ranged from 20 to 68 years old with a mean age of 36 years old. Most participants identified as cisgender women, with one person identifying as gender fluid. Half the participants (50%) had experience with a multidisciplinary pain clinic, and more than half (70%) had received pelvic PT before. Refer to Table 1 for detailed information regarding participant demographics.

**Table 1.** Participant demographic information (*n* = 10).

Characteristic	Description
Experience with multidisciplinary pain clinic	
Yes	5 (50%)
No	5 (50%)
Received pelvic physiotherapy previously	
Yes	7 (70%)
No	3 (30%)
Mean age (years)	36.1 (20–68)
Gender identity	
Cisgender woman	9 (90%)
Gender fluid	1 (10%)
Household income	
$0–19,999	1 (10%)
$20,000–39,999	2 (20%)
$40,000–59,999	1 (10%)
Prefer not to answer	5 (50%)
Highest level of education	
Secondary school	3 (30%)
College	3 (30%)
University	4 (40%)
Living status	
Rent	3 (30%)
Own	2 (20%)
Temporary housing	4 (40%)
Lodging	1 (10%)
Occupational status	
Paid employment outside the home – full time	1 (10%)
Paid employment outside the home – part time	1 (10%)
Caregiver	1 (10%)
Student	1 (10%)
Retired	1 (10%)
Medical leave	1 (10%)
Other (did not specify)	4 (40%)
Method of household income	
Paid employment	3 (30%)
Long-term disability	1 (10%)
Partner/spouse	1 (10%)
CPP disability benefit	1 (10%)
Other (did not specify)	4 (40%)

Here we describe three main themes to convey the experiences of participants living with CPP and their expectations for pelvic PT.

### Expectations are clouded by a lack of understanding

Participants’ expectations for pain management and pelvic PT were closely tied to their limited understanding of their condition and of evidence-based pain management. While most recognized the chronic nature of their CPP, few could define their condition clearly.

It’s just pain, I guess. I can’t really explain the pain. (P1, age 34)

This uncertainty was compounded by diagnostic challenges. Participants often searched extensively for a diagnosis, using online resources and consulting various healthcare providers, yet felt uncertain about the root cause. Although they identified possible contributing factors like diet, stress, weight, and physical activity, they struggled to understand how these related to their condition holistically. One participant shared their frustration:

At one point I thought that could it be my weight, but I’ve never really been obese. . . then another thing I thought [was that it] could be a lack of activities. . . [but] that wasn’t the case either because I did a lot of walking, a lot of bike riding. . . [and] at one point I thought certain types of food [contributed]. . . Everything is just so frustrating. (P2, age 52)

Regardless of previous pelvic PT experience, participants found it challenging to articulate expectations for their sessions, often responding with phrases like “I guess” and “maybe.”

I guess they will do something with my pelvic [region], probably massage. . . The other physiotherapist that I used to go to, she worked with her hands on me, and she applied pressure in certain areas. . . (P9, age 68)

Many participants reported being referred to pelvic PT without receiving clear explanations about the therapy’s purpose or its role within a biopsychosocial model of pain management. This lack of understanding created uncertainty about how pelvic PT fits into their broader pain management plans. Participants frequently expressed confusion about the goals of PT, with one noting:

Just what I’ve been taught with the exercises. . . But other than that, nothing, really. (P2, age 52)

For some participants, barriers to accessing pelvic PT further compounded these challenges. Socio-economic, physical, and systemic factors often limit their ability to engage in care. Participants described difficulties as insufficient health insurance coverage, which prevented them from continuing treatment after an initial assessment. One participant shared:

[I] was only able to do an intake portion before insurance and some other stuff took over. (P8, age 30)

These barriers were not solely financial. Geographic and systemic challenges also played a significant role, where some participants lived in areas where pelvic PT services were not readily available. This made it difficult to access care without having to travel long distances. Others reported not knowing where to find providers specializing in pelvic health, which delayed or entirely prevented their engagement with recommended treatments. Participants also highlighted a lack of clarity about how pelvic PT differs from traditional musculoskeletal PT, with which they were more familiar. This gap in understanding led to uncertainty about what to expect from pelvic PT. For instance, while one participant associated musculoskeletal PT with “different exercises, different muscle groups, different ways to move. . .” (P10, age 22), another participant described pelvic PT as “they’re just going to go through certain exercises and stretches to help with pain. . . All I know so far is that it helps you just relax [your] pelvic floor muscles, and also find ways to manage the pelvic pain too.” (P6, age 22)

These findings underscore the need for enhanced patient education to clarify the purpose, score, and benefits of pelvic PT. Additionally, addressing systemic barriers, such as limited service ability and inadequate insurance coverage, is critical to improving access and outcomes. This combination of limited understanding, financial constraints, and geographic challenges highlights the complexity of barriers faced by patients and suggests the importance of tailored interventions to support equitable care delivery.

### Pelvic PT will provide a new way to get relief

Participants shared expectations for PT in managing their CPP, focusing less on complete pain relief and more on long-term strategies to reduce pain intensity and frequency. As P2 (age 52) put it, “not going to be pain-free because there’s no cure.” Instead, participants sought methods to manage pain, as P3 (age 63) described: “How I [can] manage the pain. . . get some release. . . I know that is not going to be perfect. . . just [to] improve my situation.”

Several participants anticipated pelvic PT would provide relief and enhance their function. P9 (age 68) shared, “I find that everything I do is only. . . a band-aid solution. . . I’m hoping the physio can give me some relief. . . and education on how to manage my health. . . it’s more of an education and healing process.” However, when asked about specific treatments or self-management strategies, participants often could not identify them, with P4 (age 24) saying, “a new way to get relief, or just. . . suggestions,” and P5 (age 20) stating, “I’m not too sure.”

Participants expressed a need for a provider who understands CPP, as they often felt misunderstood within the healthcare system, contributing to a sense of isolation. P1 (age 34) explained, “When you’re trying to explain to people. . . you look healthy. . . they don’t understand. . . they don’t know what I’m feeling.” The sensitive nature of CPP also led participants to emphasize the importance of a strong therapeutic alliance with their PT, as P8 (age 30) described: “I do expect a safe space. . . confidentiality. . . encouragement to ask any questions.”

These quotes highlight patients’ expectations of pain management support, education, and a therapeutic environment where they feel genuinely heard and supported.

### My role is to be open to try new things

Participants, despite limited understanding of pelvic PT, recognized their role as active listeners and were committed to being honest and engaged in their treatment. P2 (age 52) described this commitment: “I think my role is to follow the advice, be open, be willing. And specifically, be willing to compromise, not being rigid in my thinking, and be open to new things, and new ideas.” Many participants expressed a desire for active involvement in PT, with P1 (age 34) stating, “as much as I can.” They highlighted the importance of self-management, as P3 (age 63) noted, “I think that [the] pelvic floor. . .is very related with. . . how you manage it and how often you practice with. . . exercises.”

Participants expected their pelvic PT to provide education and strategies for ongoing pain management, viewing PTs as experts who could guide them in self-care. P5 (age 20) voiced a desire for “more knowledge on. . . things that I could do for myself.” They were open to trying new techniques, hoping for pain relief and greater autonomy in managing their condition, despite feeling inadequately educated by previous providers.

Because right now I really don’t know much. And I wasn’t really told how to manage pain other than taking . . . Advil [and] Naproxen for . . . the last 7 years or so. (P6, age 22)

Participants recognized the multifaceted nature of their CPP, noting that effective management requires an “all-around. . .holistic approach” (P2, age 52). They acknowledged the need for active self-management strategies such as exercise, diet, mindfulness, and coping techniques. Although participants engaged in strategies aligning with a biopsychosocial approach, they did not explicitly identify these as biopsychosocial factors. For instance, one participant noted that “physical and emotional issues are related to each other” (P3, age 63), yet no direct mention of the biopsychosocial model was made.

Overall, participants agreed on the importance of their role in actively managing CPP, with a focus on pain relief rather than cure, and expected their pelvic physiotherapist to provide guidance on self-management techniques within a holistic framework.

## Discussion

This study aimed to understand patient expectations regarding pelvic PT for cisgender women with CPP. Three primary themes emerged: (1) Participant expectations are clouded by a significant lack of understanding of CPP and pelvic PT, (2) Pelvic PT will provide a new way of symptom relief, and (3) The patient’s role is to be open and try new things. Findings reveal a lack of understanding of CPP, emphasizing the need for education from healthcare providers. There was confusion between traditional musculoskeletal PT and pelvic PT, which led to unclear treatment expectations. Participants also expressed diagnostic uncertainty which affected their engagement with pelvic PT. Participants anticipated education from physiotherapists to better manage their pain, acknowledging an active role in PT sessions and self-management strategies, aligning with a biopsychosocial approach to care.^26^

Participants expressed confusion around CPP and its management, expecting physiotherapists to bridge these knowledge gaps. Literature indicates dissatisfaction among women with CPP when healthcare providers focus solely on physical symptoms, underscoring the importance of the biopsychosocial model in care.^27^ Although participants could identify factors like stress and diet as influences on their pain, they struggled to connect these with their pain experiences, suggesting inadequate understanding of biopsychosocial factors that influence pain. Recognizing this gap may guide future care by enhancing patient understanding of CPP’s multifactorial nature, a critical factor for patient-driven management strategies.^28^

Participants communicated that learning how to self-manage their CPP was an expectation of PT care, particularly active strategies like exercises and mindfulness. Research supports that active self-management through home exercise and relaxation techniques is effective in managing CPP.^29,30^ Providing such strategies can align patient expectations with treatment goals, improving outcomes and quality of life. Bovbjerg et al.^31^ highlight that when patient-centered goals are addressed, this increases patient satisfaction and for women with pelvic floor dysfunction. By encouraging patients to take control of their own care and adopt self-management strategies, we not only provide valuable education but also support analgesic neuroplastic changes known to improve pain outcomes.^32^

Given the complexity of CPP, patients often have trouble navigating the healthcare system, exposing themselves to misdiagnosis and feelings of isolation.^28^ Literature shows that inadequate support from providers often leads to emotional strain and distrust in healthcare.^28^ Participants expressed the need to be heard and validated in their experiences of CPP, which are often invisible to others. Validation in the early stages of treatment may enhance adherence and trust in the therapeutic relationship.^5,33^ The findings of this study show that there is value in tailoring pelvic PT treatment to champion education, self-management strategies, and providing support for patients to feel heard. Consequently, utilizing a biopsychosocial approach will foster a safe space for women with CPP, promoting active self-management and improved patient-provider alliances. This will lead to improved treatment and health outcomes for patients with CPP.

### Limitations

A primary limitation of this study is the social positionality of the participants. We attempted to recruit a diverse participant sample; however, viewpoints from underrepresented groups are limited. Only one participant identified as gender fluid, which might limit the transferability of experience findings from the entirety of gender-fluid individuals living with CPP. A second limitation of our study is the development of our interview guide. The guide was trialed within our research team instead of with patients who have experienced CPP. This may have caused us to omit critical questions that would have strengthened the depth and quality of data collected during the interviews based on lack of experience with the condition. However, during each interview, the team was able to follow up with relevant probing questions at appropriate times to stimulate further conversation based on specific patient experiences. A third limitation is that ethnicity and cultural backgrounds were not collected as part of this study. These factors have been shown to play a role in healthcare expectations, could influence participants’ responses, and could be an interesting opportunity for future study.^34^ Additionally, 70% of the participants reported receiving pelvic PT prior, which could alter PT expectations. Thus, further research should explore the impact of ethnicity and culture on PT expectations as well as focus on participants with no prior exposure.

## Conclusion

Our study found that participants’ expectations for pelvic PT were influenced by their lack of understanding of pelvic PT, which made their expectations unclear. We also found that participants expect pelvic PT to teach them new pain relief strategies and that they expect to play an active role in their treatment. Our findings revealed that it is key for pelvic physiotherapists to understand their client’s expectations for treatment, as acknowledging their client’s experiences, beliefs, and goals will allow them to tailor treatment plans to be more effective. Providing care using the biopsychosocial approach will ensure that pelvic physiotherapists offer meaningful treatment and education that will improve the success of pelvic PT care.

## Supplemental Material

sj-docx-1-whe-10.1177_17455057251349626 – Supplemental material for Where best practice pain care and patient expectations for care meet: Exploring patient expectations around chronic pelvic pain, physiotherapy, and the biopsychosocial model of care Supplemental material, sj-docx-1-whe-10.1177_17455057251349626 for Where best practice pain care and patient expectations for care meet: Exploring patient expectations around chronic pelvic pain, physiotherapy, and the biopsychosocial model of care by Michelle Hong, Allison Crone, Elza Gashi, Meghan Pietluch, Maddy Reinders, Jayden Uchida, Adriano Nella, Crystal MacKay, Olivia Drodge and Rachael Bosma in Women’s Health
