# Supplementary material for: Where best practice pain care and patient expectations for care meet: Exploring patient expectations around chronic pelvic pain, physiotherapy, and the biopsychosocial model of care
Source: Womens Health (Lond). 2025 Jun 25;21:17455057251349626. doi: 10.1177/17455057251349626 (PMC12198529; doi:10.1177/17455057251349626)
Supplement: sj-docx-1-whe-10.1177_17455057251349626 – Supplemental material for Where best practice pain care and patient expectations for care meet: Exploring patient expectations around chronic pelvic pain, physiotherapy, and the biopsychosocial model of care [file sj-docx-1-whe-10.1177_17455057251349626.docx]

**Demographic Questionnaire**

We are going to go through a series of general questions to get a better understanding of who you are. We collect this information so we can understand more about who our patient population is. The information we are collecting is from our entire study population and will therefore be a collection of data reported in averages, nothing will be reported as an individual finding. As a reminder, we want to emphasize that you do not have to answer every question. If there is something you are not comfortable with answering, just say “move on”. Do I have your consent to continue?

Participant: Yes (Continue) or No (Thank them for their time and do not continue with questionnaire)

Do you have any questions or concerns before we begin?

1. Have you ever received care before within a multidisciplinary chronic pain clinic?
2. Yes
3. No
4. Prefer not to answer

1. Have you ever received pelvic physiotherapy services before?
2. Yes
3. No
4. Prefer not to answer

1. Have you ever had surgery related to your chronic pelvic pain
2. Yes
3. If yes, when was the last surgery?____
4. No
5. Prefer not to answer

1. What is your age in years? ________

1. Which gender do you identify most with?

Please stop me when I have read the category which applies to you:

1. Cis Woman
2. Cis Man
3. Non-binary
4. Trans Woman
5. Trans Man
6. Gender Fluid
7. Two-Spirit
8. Other: ______
9. Prefer not to answer

1. What is your household income per year?

Please stop me when I have read the category which applies to you:

1. $ 0-19 999
2. $ 20 000-39 999
3. $ 40 000-59 999
4. $ 60 000-79 999
5. $ 80 000-100 000
6. > $ 100 000
7. Prefer not to answer

1. Highest level of education achieved:

Please stop me when I have read the category which applies to you:

1. No schooling completed
2. Elementary school
3. Secondary school
4. College
5. University
6. Other: ______
7. Prefer not to answer

1. Living status:

Please stop me when I have read the category which applies to you:

1. Rent
2. Own
3. Unsheltered
4. Affordable housing (government/public housing)
5. Overnight shelter/day shelter
6. Temporary housing  (including living with a friend/family)
7. Other: _______
8. Prefer not to answer

1. Occupational status:

Please stop me when I have read the category which applies to you:

1. Paid employment outside the home/full time
2. Paid employment outside the home/part time
3. Paid employment outside the home/casual
4. In home work
5. Caregiver
6. Volunteer
7. Other: ______________
8. Prefer not to answer

1. Method of household income:

Please stop me when I have read the category which applies to you:

1. Paid employment
2. WSIB
3. ODSP
4. LTD
5. Partner/spouse
6. Other: ______
7. Prefer not to answer

1. Are you currently taking an opioid to help manage your pelvic pain?
2. Yes
3. No
4. Prefer not to answer

1. How many minutes is your commute from home to TAPMI?

Please stop me when I have read the category which applies to you:

1. < 10 mins
2. 10-30 mins
3. 30-60 mins
4. > 60 mins
5. Prefer not to answer

1. What is your preferred method of transportation for this commute?
2. Walking
3. Bicycle
4. Personal vehicle
5. Public transit (bus, train)
6. Public vehicle (uber, lyft, taxi)
7. Public accessibility transport (eg: wheel-trans)
8. Other: ______
9. Prefer not to answer

1. How accessible do you perceive your commute to be? (likert scale)
2. Very accessible (5)
3. Mostly accessible (4)
4. Neutral (3)
5. Not easily accessible (2)
6. Completely inaccessible (1)
7. Prefer not to answer

**Interview Guide**

*Interviewer 1:* Hello [insert participant name], I am [insert interviewer 1 name].

*Interviewer 2:* Hello [insert participant name], I am [insert interviewer 2 name]. Thank you for taking your time to participate in this study. Just a reminder, we are meeting today to explore your thoughts around chronic pelvic pain and expectations of pelvic physiotherapy. Meghan will be facilitating the interview and I will be taking notes of your responses throughout this interview. Meghan will be asking you a series of questions but it is more like a conversation between us. Naturally, some of these questions can be sensitive in nature and I want to emphasize that you are welcome to stop and take a break at any time, ask questions at any time, and can refuse to answer any questions you don’t feel comfortable with. As we go through the interview, I will be taking notes so I just want to let you know that you may see me looking down often or hear typing in the background. Are you ready to begin?

*Participant:* Yes (continue) or No (terminate study)

*Interviewer 2:* Before we start, I will be going over important information about the study and completing the consent form with you. So first things first, do I have your consent to audio (and video) record this meeting including the consent process, demographic questionnaire, and interview?

*Participant:* Yes (continue) or No (terminate interview)

*Interviewer 2:* Thank you, I am beginning the recording now. I am now going to read a series of statements regarding consent, after I read them please state whether you consent or not to contiuing with the interview.

[Interviewer will read the **Participant Statement of Consent** statements found in Appendix E and will ask the participant to state their name and indicate that they consent]

*Interviewer 1:* Great. Now I will start with the demographic questionnaire and will share my screen so you can see the responses to the questions.

[Interviewer will complete the demographic questionnaire found in Appendix H.]

*Interviewer 1:* Thank you for completing those documents. Before we proceed with the interview, do you have any questions?

*Participant*: Yes (answer participant’s questions) No (continue to the interview)

1. What is your understanding of chronic pelvic pain?

- Probes:
  - What resources of you consult to gain information on chronic pelvic pain?

1. What do you think is contributing to your chronic pelvic pain?

- Probes:
  - Can you elaborate on that?
  - What are you doing to manage your pain?
  - What are you doing to manage the thing that is contributing to your pain?

1. You have been referred for pelvic physiotherapy. What do you know about pelvic physiotherapy?

- Probes:
  - Where do you usually go to get this information regarding pelvic physiotherapy?
  - Do you know why you have been referred for pelvic physiotherapy?

1. Have you ever received pelvic physiotherapy before?

- If participant answer yes, the following probe can be used:
  - Can you tell me more about your experience?
- Have you ever been referred to pelvic physiotherapy before?
  - Can you tell me more about that?

1. What do you think is going to happen during your pelvic physiotherapy appointment?

- Probes:
  - What do you expect to be offered?
  - Do you expect your pelvic physiotherapist to teach you anything?
  - What do you expect to learn about?
  - How long are you expecting to be involved in pelvic physiotherapy? How many sessions do you think you’ll need to help manage your pain? How long do you think your pelvic physiotherapy sessions will be?

1. What are your expectations from your physiotherapy care?

- Probes:
  - What do you expect the outcome will be?
  - Can you elaborate on that?

1. What do you hope will be the focus of your physiotherapy sessions?

- Probes:
  - What types of treatment do you feel like you need to manage your pelvic pain?
  - What information do you want to be shared with you?

1. What is your role in your physiotherapy care?
2. If you could name one goal that you want to get out of pelvic physiotherapy, what would it be?

- Probes:
  - Can you elaborate more on that?

Is there anything you wanted to revisit or discuss more about?

“Thank you very much for taking the time to participate in our study. This concludes the interview. I am going to stop the recording. Once the study results are published, would you like to receive a summary of the results?

*If yes:* Record participant email to receiving study results summary __________________.

If you have any questions, comments, or concerns, please do not hesitate to contact Dr. Rachael Bosma, rachael.bosma@wchospital.ca
